# Sumoylation of Human Argonaute 2 at Lysine-402 Regulates Its Stability

**DOI:** 10.1371/journal.pone.0102957

**Published:** 2014-07-18

**Authors:** Umut Sahin, Pierre Lapaquette, Alexandra Andrieux, Guilhem Faure, Anne Dejean

**Affiliations:** 1 Laboratory of Nuclear Organization and Oncogenesis, Institut Pasteur, Paris, France; 2 Institut National de la Santé et de la Recherche Médicale, U993, Paris, France; 3 Equipe Labellisée Ligue Nationale Contre le Cancer, Paris, France; 4 Centre National de la Recherche Scientifique, Université Pierre et Marie Curie, UMR7590, Paris, France; NIGMS, NIH, United States of America

## Abstract

Gene silencing by small RNAs has emerged as a powerful post-transcriptional regulator of gene expression, however processes underlying regulation of the small RNA pathway *in vivo* are still largely elusive. Here, we identified sumoylation as a novel post-translational modification acting on Ago2, the main effector of small RNA-mediated gene silencing. We demonstrate that Ago2 can be modified by SUMO1 and SUMO2/3 and identified Lys402 as the major Ago2 sumoylation site *in vivo*. Ago2 physically interacts with the SUMO E2 conjugating enzyme Ubc9 and the E3 ligase RanBP2 facilitates Ago2 sumoylation *in vitro*. Mutation of Lys402 enhances the stability of Ago2 protein and impairment of cellular sumoylation by siRNA- or shRNA-mediated extinction of Ubc9 or in Ubc9 knockout mouse tissues results in increased steady-state levels and enhanced stability of Ago2. Similarly, knockdown of RanBP2 or of the SAE2 E1 enzyme enhances Ago2 protein levels. Lys402 is located in the L2g1 loop linking the PAZ and PIWI domains of Ago2, in the immediate vicinity of Tyr393 which can be phosphorylated, implying that the L2g1 linker represents an easily accessible hot spot for post-translational modifications. Altogether, our results show that sumoylation of Ago2 at Lys402 negatively regulates its stability, thereby establishing a first link between SUMO and the small RNA machinery.

## Introduction

RNA silencing by small non-coding RNAs represents a key contributor to gene regulation in eukaryotes. Two major classes of small RNAs function in RNA silencing: siRNAs (small interfering RNAs) and miRNAs (microRNAs). Although cells are rarely exposed to siRNAs except mostly during viral infection, miRNA-mediated gene silencing seems to be common to all eukaryotes. miRNAs are the best-characterized class of small non-coding RNAs in mammals and are predicted to regulate over 50% of all human protein-coding genes [1]. Argonaute (Ago) proteins are the central components of the siRNA- and miRNA-containing effector complexes that execute the silencing effects [2]. Genes encoding miRNAs are transcribed to primary miRNA transcripts (pri-miRNAs) that undergo successive enzymatic maturation steps, firstly in the nucleus by the nuclear microprocessor complex containing the RNase III enzyme Drosha, and then by the RNase III enzyme Dicer in the cytoplasm to produce a mature miRNA [3]. siRNAs or miRNAs associate with the Ago proteins to form the RNA-induced silencing complex (RISC) that recognizes the target mRNA by complementary base pairing. Target mRNA silencing may be achieved through inhibition of translation, destabilization by deadenylation or endonucleolytic degradation, and the exact mechanism depends on the type of Ago protein in the effector complex, as well as on the extent of complementarity between the small RNA and the target mRNA [4]. Ago2 is the only mammalian Ago protein with intrinsic endonuclease activity (also referred to as the ‘slicer’ activity) and therefore is considered as the catalytic engine of the RNA silencing machinery [5]–[7].

Ago2 also acts at multiple levels during miRNA biogenesis. The ribonuclease Dicer has been traditionally accepted as the central processing enzyme of small RNA maturation. However, a novel miRNA processing pathway independent of Dicer, which instead requires Ago2 catalysis, has been described for at least miR-451 [8], [9]. In addition, during biogenesis of some miRNAs, Ago2 can generate an additional miRNA precursor. This so-called Ago2-cleaved precursor miRNA (ac-pre-miRNA) has been suggested to facilitate strand dissociation of the mature miRNA [10]. Beyond its fundamental role in RNA silencing, Ago2 also takes part in various biological processes requiring non coding small RNAs, including chromatin silencing, control of alternative splicing or DNA damage response [11]–[13].

The multiple roles of Ago2 during miRNA maturation and effector phase of silencing imply that this protein is likely subject to tight regulation at various levels, including its expression, stability, activity, subcellular localization or interactions with partner proteins. Reversible post-translational modifications are widely used to dynamically regulate protein activity. Several recent reports demonstrate that Ago2 is post-translationally modified by phosphorylation, ubiquitination and prolyl-hydroxylation. EGFR-dependent phosphorylation of Ago2 at Tyr393 in response to hypoxia suppresses maturation of certain tumor-suppressor miRNAs [14]. On the other hand, phosphorylation at Ser387 modulates Ago2 subcellular localization to P-bodies (processing bodies), whereas phosphorylation at Tyr529 prevents small RNA binding [15], [16]. Ubiquitination of Ago2 plays a central role in its turnover [17] and hydroxylation at Pro700 regulates Ago2 stability [18].

Covalent modification by SUMO has recently emerged as an important and highly dynamic post-translational regulator of protein function. Sumoylation has critical roles in diverse cellular processes including transcription, chromatin function, DNA repair and protein stability [19]–[22]. In vertebrates, three SUMO paralogues are expressed, designated as SUMO1, SUMO2 and SUMO3, with SUMO2 and SUMO3 nearly identical (hereafter referred to as SUMO2/3). SUMO is reversibly conjugated to its target through an enzymatic cascade involving a heterodimeric E1 activating enzyme (SAE1/2), a unique E2 conjugating enzyme (Ubc9) and one of several E3 ligases. Like ubiquitin, SUMO2/3 can form polymeric chains on target proteins through branching at a conserved lysine (Lys11). Although, unlike ubiquitination, sumoylation does not directly target its substrates to the proteasome for degradation, recent studies uncovered a cross-talk between SUMO and the ubiquitin-proteasome system involving SUMO-targeted ubiquitin ligases (STUbLs) [23].

In this report, we identify sumoylation as a novel post-translational modification regulating Ago2 stability. We demonstrate that human Ago2 can be modified by both SUMO1 and SUMO2/3 on Lys402. Mutation of Lys402, or impairment of cellular sumoylation upon transient or stable depletion of several SUMO enzymes, leads to stabilization of Ago2, implying that sumoylation at Lys402 enhances Ago2 turnover and antagonizes its stability.

## Materials and Methods

### Plasmid constructions

Ubc9, SUMO1, His-HA-SUMO1, SUMO2, and His-HA-SUMO2 expression vectors were described previously [24]. Point mutant derivatives of Ago2 (K62R, K266R, K402R, K693R, K62R/K266R/K402R/K693R quadruple mutant, E404A or Y393A) were constructed by site-directed mutagenesis (QuikChange XL kit; Stratagene).

### Antibodies and chemicals

For western blot, the following antibodies were used: rabbit anti-Ago2 (Sigma) at 1∶1000, mouse anti-HA (16B12, Covance) at 1∶2000, mouse anti-Ubc9 (BD biosciences) at 1∶1000, mouse anti-tubulin (Sigma) at 1∶10 000, in house rabbit anti-SUMO1 at 1∶1000, mouse anti-RanBP2 (Santa Cruz) at 1∶400, rabbit anti-SAE2 (Abcam) at 1∶1000, mouse anti-Ago1 (Upstate) at 1∶1000, mouse anti-Vinculin (Abcam) at 1/1000, rabbit anti-PARP1 (Santa Cruz) at 1∶1000 and mouse anti-GFP (Sigma) at 1∶500. For immunofluorescence, antibodies used are the following: rabbit anti-Dcp2 (Sigma) at 1∶300, mouse anti-HA (16B12, Covance) at 1∶500, rabbit anti-Ago2 (Abcam) at 1∶300. DAPI was purchased from Invitrogen. Cycloheximide and NaAsO_2_ solution were purchased from Sigma.

### Cells and transfections

Hela cells, Ubc9+/+ and Ubc9FL/− primary MEFs and all other cell lines were cultured in Dulbecco’s modified Eagle medium (DMEM) supplemented with 10% fetal bovine serum (FBS) and antibiotics under standard culture conditions. HeLa cells were transfected with indicated expression vectors for 48 h by using Lipofectamine 2000 (Invitrogen).

### Tamoxifen Treatment for Ubc9 ablation

8- to 12-week-old Ubc9//ROSA26-CreERT2 and Ubc9fl//ROSA26CreERT2 mice were daily injected with vehicle (corn oil) or with 1 mg 4-hydroxytamoxifen (4-OHT) [30]. For MEFs, 4-OHT was added directly in the culture medium (100 nmol/L) during 6 days.

### *In vitro* SUMOylation assay

^35^S-methionine-labeled *in vitro* translated Ago2 was prepared using the T7 TNT-coupled reticulocyte lysate kit (Promega) and incubated with recombinant E1 enzymes (SAE1/2, 370 nM), E2 enzyme (Ubc9, 630 nM) and SUMO1 or SUMO2 (7 *μ*M) in a reaction buffer (30 mM Tris, 5 mM ATP, 10 mM MgCl_2_, pH 7.5), at 30°C for 90 min, as previously described [25]. Where indicated, recombinant RanBP2 33 kDa fragment (BPΔFG), as described previously [25] was added in the reaction mix. The reaction was stopped by the addition of Laemmli sample buffer and analyzed by SDS–PAGE.

### Statistical analyses

All experiments were performed at least 3 times, in most cases a representative blot is shown. Statistical analyses were performed using two-tailed Student’s t-test to calculate p-values.

### Ethics statement

Animals were housed in the Institut Pasteur animal facilities accredited by the French Ministry of Agriculture for performing experiments on live rodents. Work on animals was performed in compliance with French and European regulations on care and protection of laboratory animals (EC Directive 2010/63, French Law 2013-118, February 6th, 2013). Full efforts were made to minimize suffering and discomfort of the animals.

## Results

### Ago2 is SUMO-modified and interacts with Ubc9

**Figure 1:**
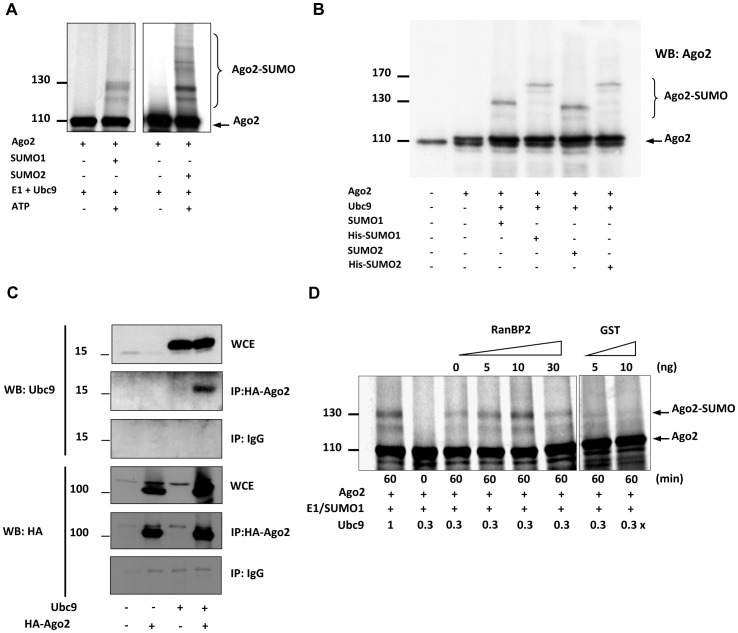
*In vivo* and *in vitro* sumoylation of human Ago2. (**A**) Ago2 is modified by SUMO1 and SUMO2 *in vitro*. ^35^S-labelled *in vitro-*translated Ago2 was incubated in a sumoylation mix containing purified SAE1–SAE2, Ubc9 and SUMO1 or SUMO2, in the absence (-) or presence (+) of ATP. Reaction products were visualized by SDS–PAGE and autoradiography. (**B**) Sumoylation of Ago2 *in vivo*. HeLa cells were transfected with expression vectors for HA-tagged Ago2, SUMO1, His-SUMO1, SUMO2 and His-SUMO2 as indicated and analyzed by Western blot by anti-Ago2 antibody. (**C**) Ubc9 interacts with Ago2 *in vivo*. HeLa cells were transfected with expression vectors for HA-Ago2 and Ubc9 as indicated. Cell lysates were immunoprecipitated (IP) with mouse anti-HA antibody or control antibody (IgG) and probed with anti-HA and anti-Ubc9 antibodies. WCE, whole-cell extract, 2% of amount used in IP. (**D**) SUMO E3 ligase RanBP2 enhances sumoylation of Ago2 *in vitro*. *In vitro* sumoylation of ^35^S-labelled, *in vitro*-translated human Ago2 in the absence or presence of RanBP2 or GST control. Reactions were incubated for indicated times (t = 0 min or t = 60 min) with 10 ng (1x) or 3 ng (0.3x) recombinant Ubc9. Reaction products were visualized by SDS–PAGE and autoradiography. Each blot is a representative of three independent experiments.

So far only limited information is available regarding post-translational modifications acting on the components of the RNA interference pathway; in particular, nothing is known about a potential role of sumoylation in this process. We therefore investigated whether Ago2, the key catalytic component of the RISC complex, was subjected to sumoylation. To this end, we first performed an *in vitro* sumoylation assay by incubating *in vitro*-translated, ^35^S-methionine-labeled Ago2 with recombinant E1 (SAE1/2) and E2 (Ubc9) enzymes together with either SUMO1 or SUMO2. Higher molecular weight (HMW) species corresponding to sumoylated Ago2 conjugates were formed in an ATP-dependant manner demonstrating that Ago2 is modified *in vitro* by both SUMO1 and SUMO2 (Figure 1A). Interestingly, *in vitro* reaction with SUMO2 led to the formation of numerous HMW Ago2 conjugates, suggesting the formation of polymeric SUMO2/3 chains on Ago2 (Figure 1A). HeLa cells were then transfected with vectors expressing either untagged or His-tagged SUMO1, along with an expression vector for HA-tagged human Ago2 (HA-Ago2). Upon co-transfection with SUMO1, Ago2 underwent a modification resulting in a size shift of about 15 kDa suggesting conjugation of a single SUMO moiety (Figure 1B). Expectedly, a slight size difference was evident between SUMO1- and His-SUMO1-conjugated forms of Ago2. Purification of His-SUMO conjugates on Ni-NTA resins, followed by a Western blot for HA-Ago2, confirmed the identity of the HMW species as SUMO-modified Ago2 forms (Figure S1 in Figures S1). Under similar overexpression conditions in HeLa cells, Ago2 was modified also by SUMO2/3 (Figure 1B). In contrast to the ubiquitin-conjugating system where E3 ligases are responsible for target recognition, conjugation of SUMO to target proteins is mediated largely by the E2 conjugating enzyme Ubc9. We thus tested whether Ago2 and Ubc9 could directly interact *in vivo*. Upon co-transfection in HeLa cells, Ubc9 was co-immunoprecipitated by an antibody against HA-tagged Ago2, but not by an isotypic control antibody, revealing physical interaction between Ubc9 and Ago2 *in vivo* (Figure 1C).

Although in many cases sumoylation is mediated directly by Ubc9, efficient conjugation requires additional E3 ligases, which may bridge Ubc9 to substrates. One such ligase, RanBP2 has been shown to enhance sumoylation of various targets including the Nuclear Body-associated SP100 protein and histone deacetylase HDAC4 [25], [26]. To test whether RanBP2 could promote sumoylation of Ago2, we reconstituted an *in vitro* modification assay with recombinant E1 (SAE1/2), E2 (Ubc9) and SUMO1 in the absence or presence of a 33 kDa fragment of RanBP2 that was previously shown to contain the E3 ligase activity (Figure 1D) [26]. As expected, Ago2 sumoylation was greatly reduced when Ubc9 concentration was lowered to 0.3x. Interestingly, this marginal level of baseline Ago2 sumoylation was significantly stimulated by addition of RanBP2, but not GST, in a dose-dependent manner (Figure 1D). Maximum reaction efficiency was achieved with 10 ng RanBP2, whereas further increasing RanBP2 concentration had a negative effect on Ago2 sumoylation, likely due to auto-sumoylation of RanBP2 that quenches available SUMO peptides as previously reported. A similar *in vitro* reaction set up using PIAS proteins, another class of SUMO E3 ligases, did not facilitate Ago2 sumoylation, demonstrating RanBP2 specificity (data not shown). Altogether, these results show that Ago2 physically interacts with Ubc9 and can be conjugated both *in vitro* and *in vivo* by SUMO1 and SUMO2/3. Moreover, Ago2 sumoylation is markedly enhanced by RanBP2 *in vitro* suggesting that, *in vivo,* RanBP2 may act as a SUMO E3 ligase for Ago2.

### Lysine 402 is the main SUMO-acceptor site on Ago2

**Figure 2:**
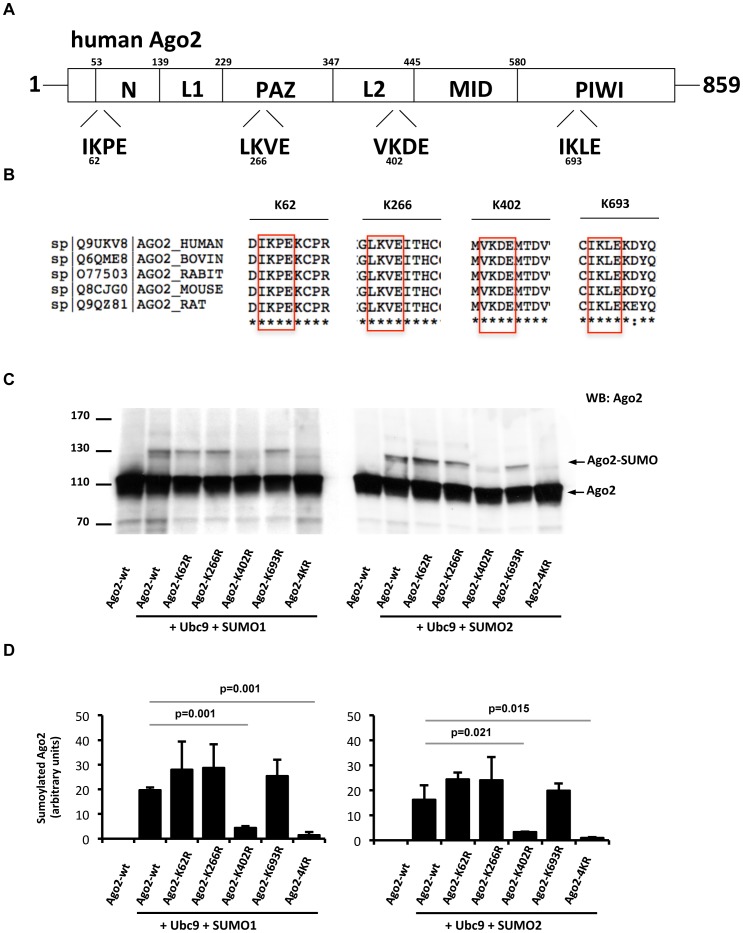
Mapping of the SUMO-acceptor sites on Ago2. (**A**) *In silico* prediction of sumoylation sites on human Ago2. Inspection of human Ago2 amino acid sequence using SUMOsp software reveals existence of four ΨKxE/D sumoylation consensus motifs. (**B**) Conservation of the four sumoylation consensus motifs (red square) on Ago2 proteins across different species. Amino acid sequences from Uniprot database were aligned using Clustalw software. (**C**) Lysine 402 of Ago2 is the major SUMO conjugation site. HeLa cells were transfected with plasmids expressing wild-type Ago2, Ago2-K62R, Ago2-K266R, Ago2-K402R, Ago2-K693R or Ago2-K62R/K266R/K402R/K693R (Ago2-4KR) in the presence of Ubc9 and either SUMO1 (left panel) or SUMO2 (right panel) and followed by Western blotting using an anti-Ago2 antibody. Representative gels from three independent experiments. (**D**) Quantification of Ago2 sumoylation in the context of wild-type Ago2 or its mutants. Extent of SUMO-conjugated Ago2 was assessed after normalization to total Ago2 levels and loading. Means and standard deviations from three independent experiments, as well as statistically significant decreases in sumoylation are shown.

Ubc9 recognizes a minimal amino-acid sequence on its target called sumoylation motif (ψKxE/D, where ψ represents a hydrophobic residue) [27]. *In silico* analysis of human Ago2 amino acid sequence indicated the presence of four such motifs (Figure 2A). These potential consensus sumoylation motifs were conserved in a variety of species ranging from mice to human, as well as between human Ago2 and Ago1 proteins (Figure 2B and Figure S2A in File S1). Of note, we found that, similar to Ago2, human Ago1 was also modified by both SUMO1 and SUMO2/3, both *in vitro* and *in vivo* (Figure S2B and C in File S1). Mutation of the lysine residues to arginines demonstrated that one of these, Lys402, was critical for Ago2 sumoylation (Figure 2C and D). Mutation of Lys402 alone (Ago2-K402R) was sufficient to abrogate SUMO conjugation to the same extent as that observed when all four putative sumoylation sites were mutated (Ago2-4KR mutant). The acidic residues immediately adjacent to the SUMO-acceptor lysines were shown to be critical for sumoylation. Importantly, mutation of Glu404 (Ago2-E404A) also significantly reduced Ago2 SUMO conjugation, demonstrating that the vicinity of K402 represents a canonical sumoylation site (Figure S1B in File S1).

Human Ago2 protein is composed of distinct structured domains, which include an N-terminal PAZ domain and a C-terminal MID/PIWI domain that are separated by a structured linker (L2) (Figure S3A in File S1). L2 is composed of L2g1, which contains an α-helix and a β-sheet, and of L2g2 which forms a small globular structure. We found that Lys402 is localized to L2g1 that connects the two halves of Ago2, and is in immediate spatial proximity of Tyr393, a residue recently reported to undergo EGFR-mediated phosphorylation [14] (Figure S3A and B in File S1). This suggests that L2g1 linker may be an easily accessible hot spot for post-translational modifications affecting, and possibly cross-regulating, various Ago2 functions. Indeed, in support of this hypothesis, we observed that Ago2 SUMO conjugation was substantially reduced when Tyr393 was mutated (Ago2-Y393A, Figure S3C in File S1).

**Figure 3:**
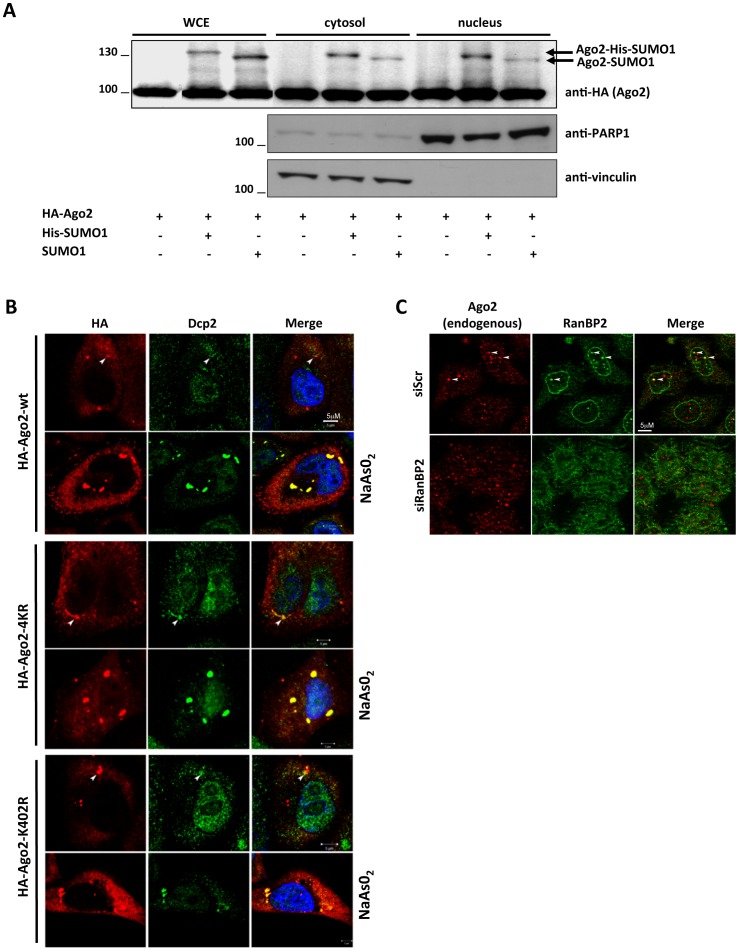
Subcellular localization of SUMO-conjugated Ago2. (**A**) Sumoylated Ago2 resides both in the cytosol and nucleus. HeLa cells were transfected with the indicated plasmids and fractionated to analyze nuclear and cytoplasmic proteins. HA-Ago2 was detected by anti-HA antibody. Immunoblots for endogenous PARP1, a nuclear protein, and endogenous vinculin, a cytoplasmic protein, were performed to validate nuclear-cytoplasmic fractionation. (**B**) Sumoylation is dispensable for subcellular localization of Ago2. HeLa cells were transfected with wild type or sumoylation mutants (4KR and K402R) of HA-Ago2, and treated as indicated (250 µM sodium arsenite -NaAsO_2_- for 60 min). Ago2 localization was determined by immunofluorescence using the anti-HA antibody (red). The cells were also immunostained for Dcp2 (marking P-bodies in green). The nuclei are stained with DAPI (blue). Ago2 staining was diffuse in the cytoplasm, as well as concentrated in cytoplasmic P-bodies (marked by arrows in untreated cells). NaAsO_2_ treatment increases size and number of P-bodies. (**C**) Endogenous Ago2 (red) and RanBP2 (green) colocalize in the nuclei of HeLa cells (indicated by arrowheads).

Sumoylation has been linked to various cellular processes including subcellular localization of target proteins and nuclear transport. Moreover, RanBP2 is located at the nuclear pore complex in vertebrates. We therefore tested whether sumoylated Ago2 was enriched preferentially in the nucleus or in the cytosol. Nucleo-cytoplasmic fractionation of cells co-transfected with expression vectors for Ago2, Ubc9 and SUMO1 indicated that the fraction of sumoylated Ago2 was nearly the same in both compartments (Figure 3A). To further investigate a potential function for sumoylation in Ago2 localization, we expressed wild-type Ago2, Ago2-K402R or Ago2-4KR mutants in HeLa cells and monitored their localization by immunofluorescence. As reported previously, Ago2 was found mostly in the cytoplasm where it partially localized to P-bodies, which have been implicated in RNA metabolism and silencing (Figure 3B). Nevertheless, mutation of Lys402 either alone or in the context of the 4KR mutant did not change this pattern (Figure 3B). Various types of cellular stress, including oxidative stress resulting from arsenic treatment, induce rapid and substantial accumulation of Ago2 in P-bodies, increasing both their size and number. Like wild-type Ago2, Ago2-K402R and Ago2-4KR were recruited efficiently to P-bodies in response to arsenic treatment (Figure 3B). These results imply that sumoylation of Ago2 is not critical for its subcellular localization, without or during oxidative stress. Importantly, Ago2 colocalized with RanBP2 in distinct nuclear foci, further supporting that RanBP2 may physically interact with Ago2, acting as an E3 ligase to enhance its sumoylation (Figure 3C).

### Impaired sumoylation enhances Ago2 stability

**Figure 4:**
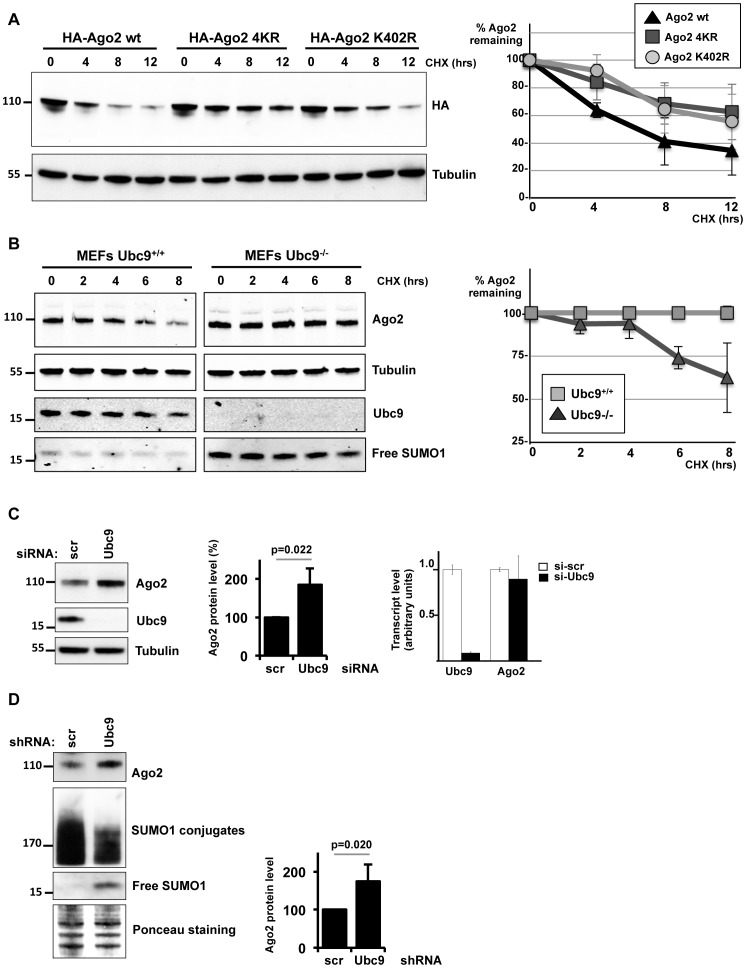
Sumoylation negatively regulates Ago2 turnover. (**A**) Sumoylation-deficient Ago2 mutants show increased half-lives. HeLa cells transfected with HA-Ago2-wt, HA-Ago2-4KR or HA-Ago2-K402R were treated with 50 µg/mL of cycloheximide (CHX) for the indicated times. The blot shown is a representative of four independent experiments. The cumulative results of are displayed on the graph (right panel). The initial levels of wild-type Ago2, Ago2-4KR and Ago2-K402R were normalized to 100%. Means and standard deviations are indicated (n = 4). (**B**) Endogenous Ago2 displays enhanced stability in Ubc^−/−^ MEFs in which global sumoylation is impaired. The blot shown is a representative of three independent experiments. CHX treatment and quantifications were performed as in A. Western blots for tubulin, Ubc9 and unconjugated SUMO1 are shown as controls. (**C**) Transient knockdown of Ubc9 results in enhanced Ago2 protein levels. HeLa cells were transfected with either a control siRNA (si-scr) or a siRNA targeting Ubc9 (si-Ubc9). Ago2 relative expression to tubulin is presented in the middle graph and normalized to 100% for siRNA-scr transfected cells. Means of three independent experiments. Graph on the right shows that Ubc9 knockdown has no effect on Ago2 mRNA expression. Total RNA extracted from HeLa cells transfected with si-scr or si-Ubc9 were analyzed by qRT-PCR with primers specific for Ubc9 and Ago2 mRNA. (**D**) Stable knockdown of Ubc9 results in enhanced Ago2 protein levels. Human HT1080 cells transfected with a control shRNA (scr) or a shRNA against Ubc9 were puromycin selected and processed for immunobloting with anti-Ago2 and anti-SUMO1 antibodies. Ago2 relative expression was quantified. Graph represents means of three independent experiments.

**Figure 5:**
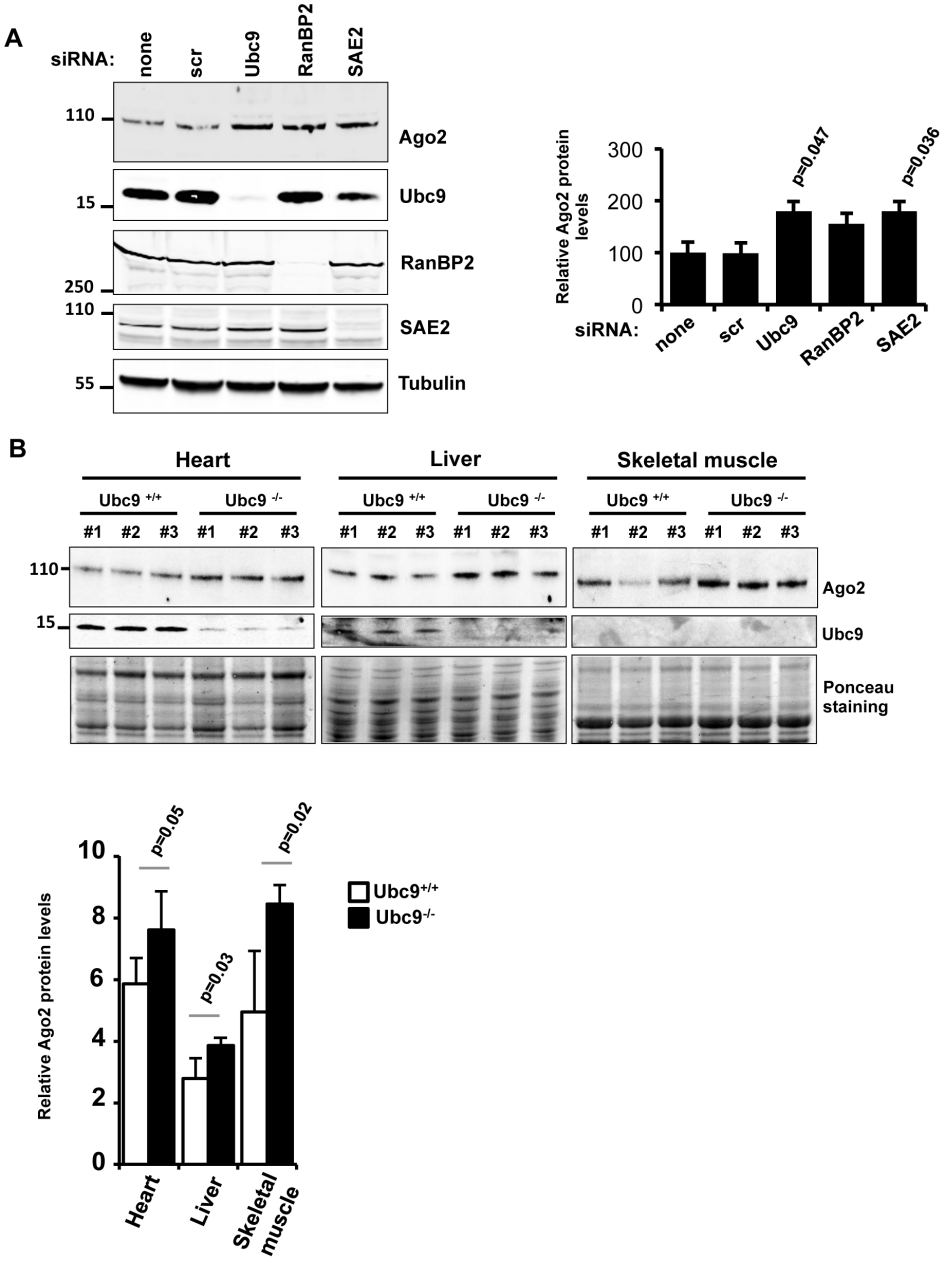
Impaired global sumoylation enhances Ago2 protein levels in cells and in mice (**A**) Depletion of SUMO enzymes results in increased steady-state Ago2 protein levels. HeLa cells were transfected with control siRNA (scr) or siRNA against Ubc9, SAE2 or RanBP2. Relative levels of Ago2 are indicated in the graph. Means of three independent experiments. Significant p-values are shown. (**B**) Loss of Ubc9 *in vivo* results in enhanced endogenous Ago2 protein levels. *Ubc9^+/+^/ROSA26-CreERT2* control (Ubc9^+/+^) and *Ubc9^fl/−^/ROSA26-CreERT2* conditional knockout (Ubc9^−/−^) adult mice (n = 3, #1, #2, #3) received 3 consecutive days of 4-OHT injection. Proteins were extracted from heart, liver and skeletal muscle and were analyzed by Western blot. Quantification of Ago2 protein levels in organs after normalization to the ponceau staining is shown. Bars represent means and standard deviations. from 3 mice (white bars: Ubc9^+/+^, black bars: Ubc9^−/−^). P-values, determined by two-tailed Student’s t-test, are indicated.

Several studies have implicated sumoylation in regulation of stability of its target proteins [28], [29]. In order to test whether sumoylation may impact on Ago2 stability, we first compared the half-lives of wild-type Ago2, Ago2-K402R and Ago2-4KR by performing cycloheximide (CHX) time-course experiments upon transfection into HeLa cells. Both Ago2-K402R and Ago2-4KR mutants were more stable (t_1/2_>12 h) than wild-type Ago2 whose half-life was estimated to be approximately 6 h, suggesting that Lys402 is important for Ago2 stability (Figure 4A). To determine whether the increased stability of the Ago2-K402R and Ago2-4KR mutants was due to loss of sumoylation, we analyzed the stability of endogenous Ago2 in MEFs isolated from wild type or Ubc9^−/−^ embryos. Importantly, endogenous Ago2 protein was more stable with significantly enhanced half-life in Ubc9^−/−^ MEFs compared to in wild type counterparts (Figure 4B). Next, we checked the steady-state levels of Ago2 in HeLa cells in which the expression of Ubc9 was inhibited by siRNA *versus* control cells. We found that Ubc9 knockdown resulted in a dramatic increase (nearly two-fold) in the steady-state level of Ago2 protein (Figure 4C), without affecting its mRNA level (Figure 4C). Similarly, human HT1080 cells impaired for global SUMO conjugation due to inhibition of Ubc9 expression by short hairpin RNA (shRNA) displayed increased levels of Ago2 protein, again by approximately two-fold (Figure 4D). In keeping with our observations upon Ubc9 knockdown, siRNA extinction of SAE2 or RanBP2 greatly enhanced (nearly two-fold) steady-state levels of Ago2, further corroborating that sumoylation antagonizes Ago2 stability (Figure 5A) and that RanBP2 may act as a SUMO E3 ligase on Ago2. We finally examined steady state levels of the endogenous Ago2 protein in mouse tissues upon *in vivo* deletion of Ubc9. *Ubc9^+/+^/ROSA26-CreERT2* control and *Ubc9^fl/−^/ROSA26-CreERT2* conditional knockout adult mice were given 3 days of 4-OHT injection [30]. Tissue lysates from heart and liver, which display moderate expression of Ubc9 and from skeletal muscle which shows low level of Ubc9 in control animals were subjected to SDS-PAGE and immunoblotting to determine endogenous Ago2 and Ubc9 levels. All three organs showed increased Ago2 protein levels upon acute ablation of Ubc9 (Figure 5B). Taken together, these findings indicate that sumoylation of Ago2 at Lys402 negatively regulates its stability.

### Impairment of sumoylation does not alter RNA interference

The observation that Ago2 sumoylation regulates its stability suggests that sumoylation may play a role in cellular RNA interference function. To address this point, we compared the siRNA- and miRNA-programmed RISC activity in cells in the presence or absence of Ubc9. To this end, we used GFP reporters containing perfect or bulged complementary sequences for miRNAs let-7 or miR-21 respectively [18]. Upon transfection into HeLa cells, the expression of GFP-let-7 or GFP-miR-21 was greatly diminished with respect to wild-type GFP, reflecting baseline cellular siRNA- or miRNA-guided RISC activity (Figure S4A and B in File S1). However, we did not detect any changes in RISC function in cells transfected with siRNA Ubc9 using this assay (Figure S4A and B in File S1). Finally, cells depleted for endogenous Ago2 by an siRNA targeting the 3′ untranslated region of Ago2 and complemented with Ago2-K402R or Ago2-4KR mutants did not display detectable changes in siRNA- or miRNA-guided RISC activity when compared to cells complemented with wild-type Ago2 (Figure S4C, D and E in File S1).

## Discussion

RNA interference pathway is a powerful and widespread gene regulatory mechanism in eukaryotes, yet much remains to be learned about cellular pathways that contribute to the regulation of its key enzymes [31], [32]. Here we show that human Ago2, the central catalytic enzyme that executes the silencing effects of small RNAs, is subject to modification by SUMO on Lys402. Mutation of Lys402 enhances stability of Ago2. Similarly, ablation of sumoylation enzymes SAE2, Ubc9 and RanBP2 increases Ago2 steady-state levels and/or stability in cells and in mice. These data imply that sumoylation of Lys402 antagonizes Ago2 stability.

Recent reports established a biochemical link between SUMO and ubiquitin pathways where STUbLs antagonize stability of sumoylated substrates. STUbLs selectively recognize sumoylated target proteins through SUMO-interacting motifs (SIM), promoting their ubiquitination and subsequent degradation. For example, RNF4 promotes SUMO-dependent ubiquitination and degradation of the tumor suppressor PML protein [33], [34], as well as of PARP1 [24]. Similarly, Arkadia, a ubiquitin ligase that upregulates TGF-β signaling by inducing the degradation of inhibitor SnoN/Ski, can also promote SUMO-dependent ubiquitination of PML [35]. We have not observed a convincing effect of RNF4 controlling Ago2 stability or its ubiquitination state (data not shown). Detailed future studies should clarify the potential role of STUbLs in SUMO-mediated destabilization of Ago2.

The small RNA binding PAZ domain of Ago2 is located above a crescent-like base structure formed by the N-terminal, middle and PIWI domains, and is separated from the base by the structured linker L2g1. Lys402 is located within this linker region and is structurally very close to part of the PIWI box that is exposed on the surface and that mediates binding to Dicer. This suggests that Lys402 sumoylation, in addition to regulating Ago2 stability, could also have an effect on Dicer binding. On the other hand, it is tempting to speculate that binding of Dicer to Ago2 may sterically affect accessibility of Lys402 to sumoylation enzymes, in turn influencing Ago2 stability. Tyr393, which is phosphorylated in hypoxia, is also located in the L2g1 linker region, and when phosphorylated, reduces Dicer binding [14]. Phosphorylation at Tyr393 could impact on Lys402 spatial position or accessibility. In line with this hypothesis, we have observed significant reduction in the extent of Ago2 sumoylation when Tyr393 was mutated (Figure S3 in File S1). Future studies will be necessary to assess biochemical consequences of this potential cross-regulation between Tyr393 phosphorylation and Lys402 sumoylation, as well as its structural and mechanistic basis.

Although we have not detected an effect of Lys402 mutation or of impaired cellular sumoylation on siRNA- or miRNA-guided RISC activity as determined by GFP reporter assays, the possibility remains that other aspects of RNA-mediated gene silencing, as analysed in different settings, may be affected by sumoylation. Indeed, Ago2 which has a preference for siRNA-initiated endonucleolytic cleavage, mediated almost complete silencing of the siRNA-based let-7-GFP reporter (Figure S4D in File S1). This makes it difficult to assess potential effects of *‘more stable’* Ago2 on let-7-GFP (either in the context of K402R- and 4KR-Ago2 or upon impaired cellular sumoylation) which, in theory, is expected to further enhance cellular siRNA function. Yet, the latter is already baseline maximal in our settings. Future studies should determine whether Lys402 sumoylation could impact on Ago2 three-dimensional architecture or on the ability of Ago2 to recruit proteins forming the RISC complex, in turn affecting siRNA or miRNA loading to Ago2 and/or leading to altered siRNA- or miRNA-guided RISC function.

Despite our efforts, we were unable to detect a substantial amount of endogeneous SUMO-modified Ago2 in untreated cells or cells undergoing stress such as γ-irradiation or arsenic treatment (data not shown). This implies that sumoylation of Ago2 may occur either transiently in response to a yet unidentified signal/stress, and/or affects only a very small fraction of Ago2. Indeed, detecting endogenous sumoylation, especially by SUMO1, is technically challenging. *In vivo*, unconjugated SUMO1 is limiting and exists almost entirely conjugated to high-affinity targets such as RanGAP1, implying that endogenous *de novo* sumoylation, particularly by SUMO1, involves molecular competition. In most cases only a tiny fraction of target proteins is subjected to sumoylation. The sumoylated fraction may be limited to a specific sub-cellular compartment or to a specific cell type/tissue. Finally, sumoylation is a highly dynamic and transient process representing a constant competition between SUMO-conjugating and deconjugating enzymes. In line with these arguments, sumoylation of HP1α has recently been shown to be highly transient and SUMO-modified HP1α was found to be exclusively enriched at pericentric heterochromatin [36], highlighting the difficulty of detecting endogenously sumoylated fraction in the whole pool of a given substrate. The evolutionary conservation of the SUMO-acceptor lysines on Ago2 proteins across various species (Figure 2B), as well as between Ago1 and Ago2 proteins (Figure S2A in File S1), point to the importance of Ago2 sumoylation in endogenous settings. In this context, it will be interesting to determine the fraction of endogenous Ago2 modified by SUMO in different sub-cellular locations and protein complexes, in response to stress or pathological conditions.

Several lines of evidence suggest that the SUMO E3 ligase RanBP2 may be important in fine-tuning Ago2 sumoylation and stability in vivo. RanBP2 enhances Ago2 sumoylation in vitro (Figure 1D), colocalizes with Ago2 in the nucleus (Figure 3C) and antagonizes Ago2 steady-state protein level (Figure 5A). Importantly, we have not observed a significant effect of PIAS family of E3 ligases on Ago2 sumoylation (data not shown) suggesting that Ago2 SUMO conjugation may specifically be favored by RanBP2 *in vivo*.

Ubc9 is upregulated in an increasing number of malignancies such as melanoma, breast cancer and ovarian carcinoma, representing a potential target for cancer therapy [37]. In line with our data, a recent report demonstrated that melanoma cells display strong reduction of Ago2 expression on the protein level, while the mRNA level is unchanged, implying post-translational control [38]. Future studies should evaluate contribution of hyper-sumoylation to Ago2 protein reduction in melanoma and in other malignancies, which likely contributes to deregulation of miRNA expression in cancers.

The interconnection of post-translational modifications has emerged as an essential mechanism that governs the spatio-temporal regulation of signaling pathways [39]. Moreover, their reversible nature constitutes a versatile mechanism to adapt to most challenges faced by eukaryotic cells. Our results, which identify sumoylation as a novel post-translational modification acting on Ago2 add another layer of complexity to post-translational regulation of the RNA interference machinery. This may be the first sign of a complex RNA interference-SUMO interactome. Future studies should determine whether other enzymes of the siRNA/miRNA pathway are subject to sumoylation, and also reveal potential cross-talks between these modifications to regulate RNA interference.

## Supporting Information

File S1 **This file includes Methods S1 and Figures S1 to S4.**
(PDF)
