# Supplementary material for: Sumoylation of Human Argonaute 2 at Lysine-402 Regulates Its Stability
Source: PLoS One. 2014 Jul 18;9(7):e102957. doi: 10.1371/journal.pone.0102957 (PMC4103873; doi:10.1371/journal.pone.0102957)
Supplement: File S1 — This file includes Methods S1 and Figures S1 to S4. (PDF) [file pone.0102957.s001.pdf]

SUPPORTING INFORMATION

1) SUPPLEMENTARY METHODS:

Gene expression analyses

Total RNA was extracted from cultured cells using Trizol reagent (Invitrogen) according to the standard procedures. For Ubc9 and Ago2 transcript level analysis, total RNA was reverse transcribed using Applied Biosystems high capacity cDNA kit. Ago2 and Ubc9 expression levels were normalized to EF1/Ezrin expression.

Determination of Ago2 stability

For measurement of Ago2 half-life, cells were treated for up to 24 hrs with cycloheximide (CHX) at a concentration of 50 μg/mL. Cells were harvested at indicated times after treatment, lysed in Laemmli buffer and analysed by SDS–PAGE. The intensity of each band was evaluated by densitometry using the Image J software and Ago2 expression level was normalized to tubulin expression.

Nucleo-cytoplasmic fractionation

PBS-washed cell pellets were resuspended in 10 volumes of buffer 1 (0.5 M sucrose, 15 mM Tris [pH 7.5], 60 mM KCl, 0.25 mM EDTA, 0.125 mM EGTA, Protease inhibitor cocktail tablet [Roche]). The cells were then allowed to recover for 5 min at 4°C, 10% NP-40 was added dropwise. The reaction was stopped by adding NP-40 when the nuclei were free of cytoplasmic contaminants. The lysed cells were centrifuged at 1,000 g. The supernatant (cytoplasmic fraction) was decanted, and the pellet (corresponding to the nuclei) was resuspended in extraction buffer (20 mM Tris-Cl [pH8.5], 10% glycerol, 2 mM dithiothreitol, 0.8 M KCl). The DNA was sheared by passage through a 25-gauge syringe and incubated for 45 min on ice. The mixture was centrifuged at 21,000 g, and the nuclear fraction was dialyzed against 150 mM KCl in extraction buffer. The various cellular fractions were analyzed by Immunoblotting.

Immunofluorescence

HeLa cells or MEFs were seeded on coverslips. Cells were fixed with 3.7% paraformaldehyde in PBS for 10 min at room temperature and subsequently permeabilized by 0.1% Triton X-100. The samples were then incubated overnight at 4°C with indicated primary antibodies.

After three washes with 0.1% Tween-20 in PBS, coverslips were incubated with DAPI, and secondary antibodies conjugated with Alexa Fluor 488 or 594, and Hoechst 33258 (Molecular Probes). Microscopic analyses were carried out by semi-confocal Apotome microscopy (Axiovert 200M, Zeiss) using AxioVision software (Zeiss).

RNA interference

Ubc9, RanBP2 and SAE2 expression was ablated by an siRNA pool (Dharmacon, On-target Plus). siRNAs (50 nM final concentration) were introduced into HeLa cells using Lullaby transfection reagent (OZ Biosciences) according to the manufacturer's instructions. Cells were analysed after 72h. Human HT1080 cells were transfected with a control shRNA (scr) or a shRNA against Ubc9 and selected by puromycin as described previously [22].

Immunoblotting and Immunoprecipitations

Adult mice organs were dissected and homogenized in 1ml of lysis buffer (50mM Tris-HCl pH8.0, 0,1m M EDTA, 200 mM NaCl, 1% NP40, 10% glycerol, 20mM NEM, 1x Protease inhibitor cocktail tablets [Complete, Roche]). Proteins from cells in culture were extracted directly in Laemmli sample buffer, boiled for 5min and sonicated. Proteins from organs and cell lines were resolved by SDS-PAGE (Criterion XT bis-tris gel, BioRad), and transferred onto nitrocellulose membranes (Trans-blot turbo, BioRad). Membranes were saturated (Tropix i-block, Applied Biosystems) and incubated overnight at 4°C with the indicated primary antibodies followed by fluorescence-conjugated secondary antibodies. Immunoreactivity was visualized using an infrared imaging system (Odyssey; LI-COR Biosciences). Protein quantification was performed using Odyssey and ImageJ (National Institutes of Health) software.

For co-immunoprecipitation of Ago2 and Ubc9, cells were scraped in PBS and lysed in immunoprecipitation (IP) buffer (50 mM Tris, pH 8.0, 0.5% NP-40, 200 mM NaCl, 0.1 mM EDTA, 10% glycerol, and protease inhibitors (Complete EDTA-free, Roche)) supplemented with 10mM N-ethylmaleimide (NEM). Lysates were subsequently incubated for 2h at 4°C with anti-HA antibody and immune complexes were collected by incubation for 2h at 4°C with Protein A/G agarose beads (Calbiochem) and washed three times in lP buffer. The agarose beads were boiled for 5min in 2X Laemmli sample buffer and analysed by SDS–PAGE.

Endogenous miRNA-guided si/miRISC activity assay

The assay was performed as described in [18]. pcDNA3–GFP, pcDNA3–GFP–let-7 (PE) and pcDNA3–GFP–miR-21 (8×BU) were a kind gift from Y. Shi. Briefly, GFPL and GFP–let-7 or GFP–miR21 expression plasmids were transfected in HeLa cells that were previously transfected with siRNA (scr or against Ubc9) or HA-Ago2 (wild type or mutants) expression plasmids. Cell extracts were analysed by Western blot for GFP expression. For each condition, silencing efficiency was calculated as the percent decrease in GFP expression (from let-7-or miR-21-regulated fusions) compared to unregulated long GFP (GFPL) and control GFP (ctl).

2) SUPPLEMENTARY FIGURES:

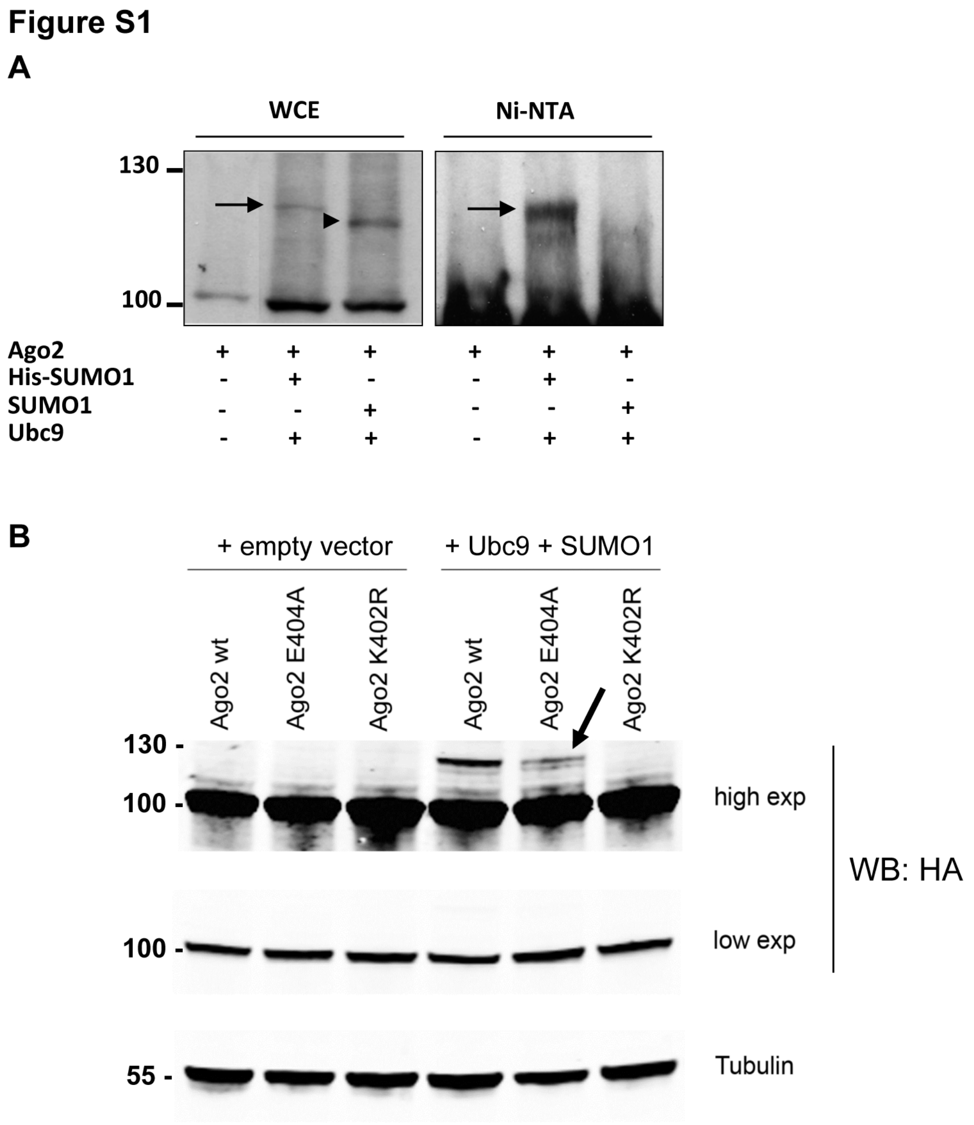

Supplementary Figure S1. Confirmation of Ago2 sumoylation *in vivo* upon purification of His-SUMO-conjugates on Ni-NTA resins and abrogation of sumoylation of the E404A mutant. (A) HA-Ago2 was co-transfected in HeLa cells with the indicated expression vectors. Whole cell extracts (WCE) or precipitates of Nickel-affinity chromatography (Ni-NTA) were analysed by western blot with anti-HA antibody. Arrows mark His-tagged SUMO-Ago2 conjugates which are enriched by Ni-affinity chromatography, while the arrowheads indicate untagged SUMO-Ago2 conjugates. **(B)** HeLa cells were transfected with

HA-tagged wild type Ago2, Ago2 E404A mutant or Ago2 K402R mutant, along with the indicated plamids. Arrow indicates impaired sumoylation of the Ago2 E404A mutant. A high and a low exposure of the same blot are shown.

Figure S2

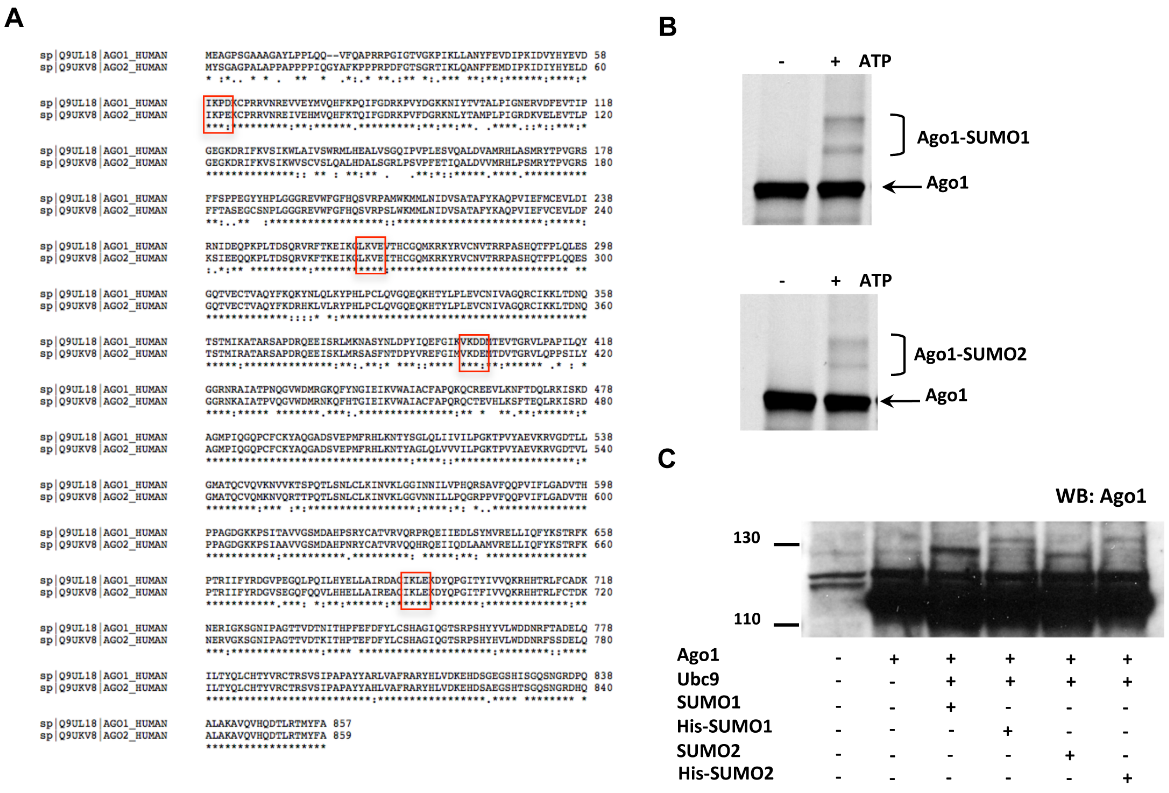

Supplementary Figure S2. Human Ago1 protein is subjected to sumoylation *in vitro* and *in vivo*. (A) Amino acids sequence alignment (clustalW) of Ago1 and Ago2 shows the conservation of the four putative sumoylation sites (red squares) between Ago1 and Ago2. **(B)** Ago1 is sumoylated *in vitro*. Sumoylation of *in vitro*-translated, ^{35}S-labeled human Ago1 with SUMO1 (upper panel) or SUMO2 (lower panel) after a 30 min reaction in the absence (-) or presence (+) of ATP in a reaction mix containing recombinant SUMO E1 and E2 enzymes. Reaction products were detected by autoradiography. **(C)** Sumoylation of Ago1 *in vivo*. FLAG-Ago1 was co-transfected in HeLa cells along with Ubc9 and various SUMO

expression vectors (SUMO1, His-SUMO1, SUMO2 and His-SUMO2) for 48 hrs. Whole cell extracts were analysed by western blot with an anti-Ago1 antibody.

Figure S3

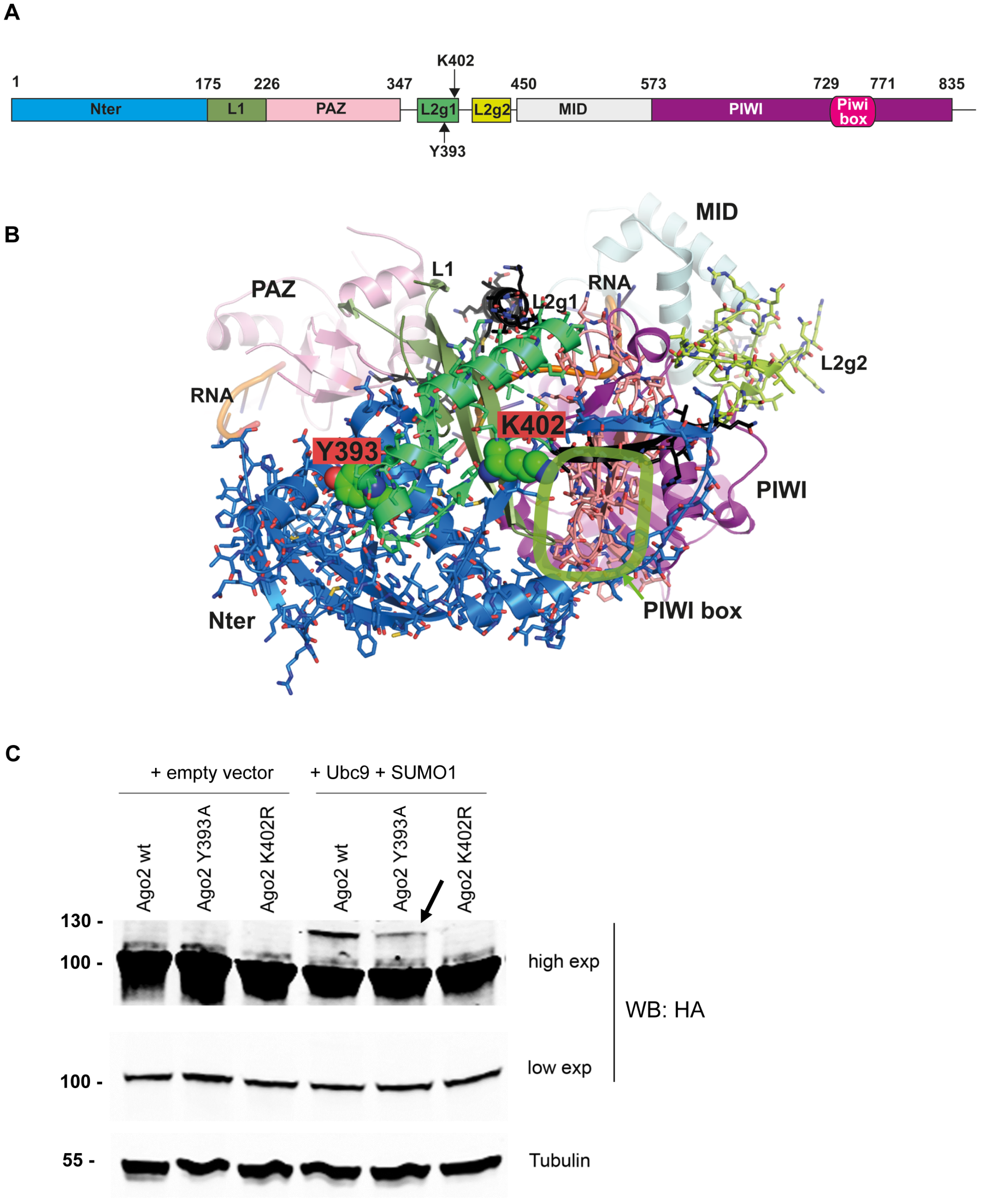

Supplementary Figure S3. Structural analysis of human Ago2. (A) Domain architecture

of the human Ago2 protein. Large boxes denote individual domains while small boxes indicate structured linkers, L1 and L2 subdivided into L2g1 and L2g2. Both Lys402 (sumoylation target) and Tyr393 (phosphorylation target) are located in L2g1. **(B)** Structure of Ago2 loaded with RNA (pdb: 4ft3) in ribbon representation. Color code corresponds to the domains described in part (A). Lys402 and Tyr393 are represented by atomic balls. The green circle shows the largest accessible surface of the PIWI box, implying the likely Dicer-binding site. **(C)** Impaired sumoylation of Ago2 Y393 mutant, as indicated by the arrow, suggests potential cross-talks between Lys402 and Tyr393 modifications. HeLa cells were transfected with HA-tagged wild type Ago2, Ago2 Y393A mutant or Ago2 K402R mutant, along with the indicated plamids. A high and a low exposure of the same blot are shown.

Figure S4

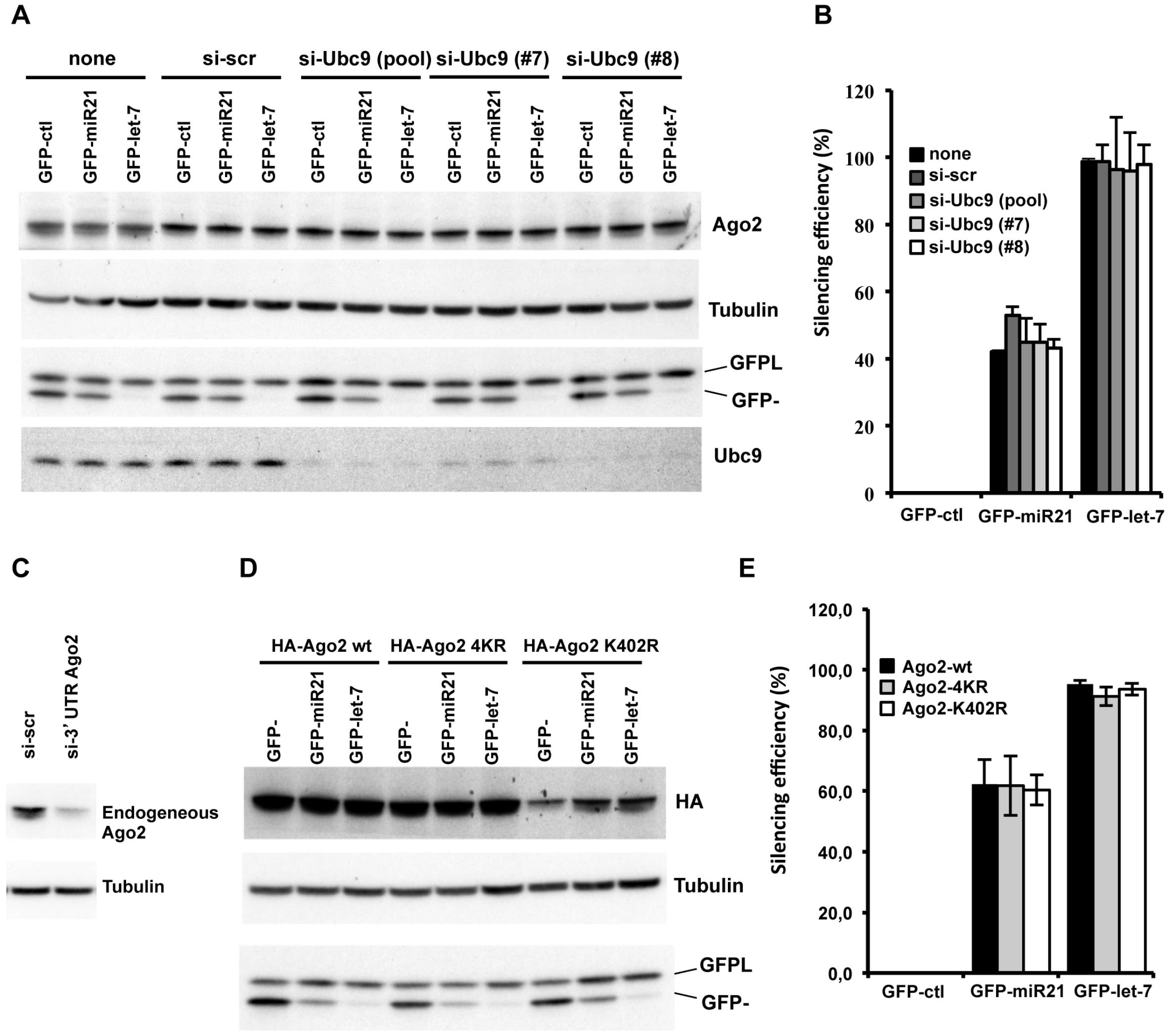

Supplementary Figure S4. Sumoylation does not affect cellular RISC activity. (A) Ago2-mediated miRISC or siRISC activity is not impaired in Ubc9 deficient cells. HeLa cells transfected first with a control siRNA (scr) or various siRNAs against Ubc9 were re-transfected with GFPL and a GFP reporter containing complementary binding sites for endogenous miR-21 (GFP-miR21) or let-7 (GFP-let-7). Cell extracts were analysed by western blot for GFP and Ubc9 expression. **(B)** For each condition, silencing efficiency was calculated as the percent decrease in GFP expression from si- (let7) or mi-RISC (miR-21) regulated reporters compared to unregulated GFP. **(C)** Immunoblot using anti-Ago2 antibody demonstrating the efficiency of the siRNA against 3'UTR of endogeneous Ago2 in HeLa

cells. **(D)** Both Ago2 and Ago2-4KR can mediate siRISC- or miRISC-guided silencing at similar extents. HeLa cells transfected first with an siRNA against 3'UTR of endogeneous Ago2 were re-transfected with either wild type Ago2 or Ago2 mutants, as well as the GFP reporters as described in A. Cell extracts were analysed by western blot for GFP and Ubc9 expression. **(E)** Quantification of the silencing efficiency corresponding to D.
